# Innexin gap junctions in nerve cells coordinate spontaneous contractile behavior in *Hydra* polyps

**DOI:** 10.1038/srep03573

**Published:** 2014-01-07

**Authors:** Yasuharu Takaku, Jung Shan Hwang, Alexander Wolf, Angelika Böttger, Hiroshi Shimizu, Charles N. David, Takashi Gojobori

**Affiliations:** 1 Hamamatsu University School of Medicine, Department of Biology, 1-20-1 Handayama, Higashi-ku, Hamamatsu, 431-3192, Japan; 2 Center for Information Biology-DNA Data Bank of Japan, National Institute of Genetics, Mishima 411-8540, Japan; 3 Helmholtz Zentrum München - German Research Center of Environmental Health, Institute of Molecular Toxicology and Pharmacology, Ingolstädter Landstr. 1, 85764 Neuherberg, Germany; 4 Department Biologie II, Ludwig Maximilians University, Munich 80539, Germany; 5 Department of Developmental Genetics, National Institute of Genetics, Mishima 411-8540, Japan; 6 Biological and Environmental Science and Engineering Division and Computational Bioscience Research Center, KAUST, Thuwal 23955-6900, Kingdom of Saudi Arabia; 7 These authors contributed equally to this work.

## Abstract

Nerve cells and spontaneous coordinated behavior first appeared near the base of animal evolution in the common ancestor of cnidarians and bilaterians. Experiments on the cnidarian *Hydra* have demonstrated that nerve cells are essential for this behavior, although nerve cells in *Hydra* are organized in a diffuse network and do not form ganglia. Here we show that the gap junction protein innexin-2 is expressed in a small group of nerve cells in the lower body column of *Hydra* and that an anti-innexin-2 antibody binds to gap junctions in the same region. Treatment of live animals with innexin-2 antibody eliminates gap junction staining and reduces spontaneous body column contractions. We conclude that a small subset of nerve cells, connected by gap junctions and capable of synchronous firing, act as a pacemaker to coordinate the contraction of the body column in the absence of ganglia.

Nerve cells and spontaneous coordinated behavior first appeared near the base of animal evolution in the common ancestor of cnidarians and bilaterians^1^,^2^,^3^. Experiments on the cnidarian *Hydra* have demonstrated that nerve cells are essential for this behavior, since removal of nerve cells leads to loss of most spontaneous behavior^4^. Nevertheless, it has been unclear how nerve cells coordinate behavior because nerve cells in *Hydra* are organized in a diffuse nerve net and do not form ganglia. We show here that a small group of nerve cells in the peduncle of *Hydra* is coupled via gap junctions thereby permitting synchronous firing^5^ and subsequent coordinated activation of effector epitheliomuscle cells.

Gap junctions are formed by innexins in a wide range of invertebrates, including the model organisms *Drosophila* and *Caenorhabditis*. Multiple innexins have been identified in the *Hydra* genome and innexin-1 has been shown to form gap junctions in ectodermal epithelial cells^6^,^7^. We now show by in situ hybridization that innexin-2 is expressed in a small group of nerve cells in the lower body column of *Hydra* and that an anti-innexin-2 antibody binds to gap junctions in the same region. Treatment of live animals with innexin-2 antibody eliminates gap junction staining and strongly reduces spontaneous body column contractions. We conclude from these results that a small subset of nerve cells in the lower body column of *Hydra*, connected by gap junctions and capable of synchronous firing, act as a pacemaker to coordinate the contraction of the body column in the absence of ganglia.

## Results

### Innexin-2 is expressed in gap junctions in nerve cells in the peduncle of Hydra

**Figure 1:**
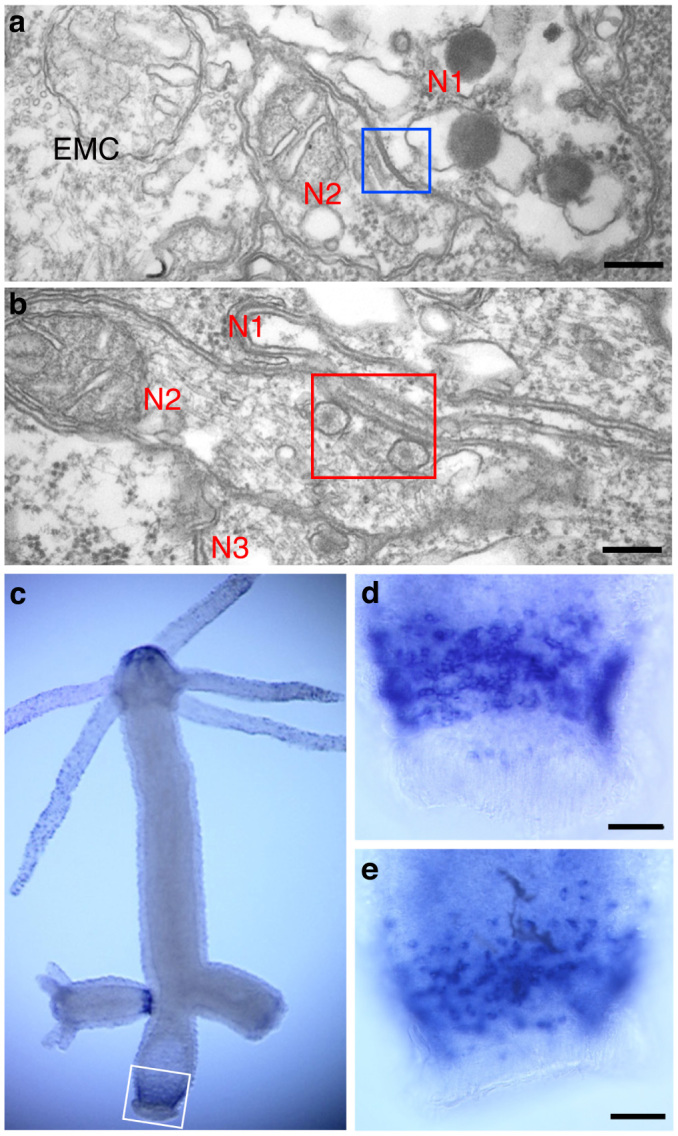
Synapses between nerve cells and expression of innexin-2 in the peduncle of *Hydra*. (a) and (b) TEM images of an electrical synapse (a) and a chemical synapse (b) between nerve cells in the peduncle. Squares outline the synapses. N1, N2, N3: nerve cells; EMC: epitheliomuscle cell. Scale bar, 200 nm. (c–e) Expression pattern of innexin-2 determined by whole mount in situ hybridization. The white box delineates the region of innexin-2 expression in the lower peduncle of the adult polyp. The fully developed bud on the left side also contains innexin-2 positive cells whereas the immature bud on the right side has not yet differentiated innexin-2 nerve cells. Staining in the tip of the hypostome appears to be background and is not associated with cellular structures. Enlarged peduncle regions of two additional polyps are shown in (d) and (e). Scale bar, 0.1 mm.

In *Hydra* large gap junction plaques are present between ectodermal epithelial cells and between endodermal epithelial cells^8^,^9^ and these junctions have been shown to mediate dye coupling and electrical coupling^10^. Gap junctions are also formed between ectoderm and endoderm via thin cytoplasmic tubules connecting epithelial cells across the mesoglea^8^. Finally, gap junctions have been documented between nerve cells in *Hydra*, although the reported numbers were small^11^. We have screened EM thin sections for gap junctions between nerve cells and found examples in all parts of *Hydra*. Figure 1A shows a typical nerve-nerve gap junction in peduncle tissue, which will be the focus of this report. The gap junctions varied from 200–500 nm in size. Numerous chemical synapses between nerve cells were also found in the same thin sections (Figure 1B). These exhibit closely apposed synaptic membranes with 150 nm dense-cored vesicles on the presynaptic side^12^,^13^.

Gap junctions in invertebrate animals are formed by innexins, which are membrane proteins with four transmembrane domains forming two extracellular loops containing conserved cysteine residues^14^,^15^. Six innexin monomers form a hemi-channel in the cell membrane and two hemi-channels on neighboring cells interact to form a gap junction channel. Innexins have been found in a large number of protostomes including *Drosophila* and *Caenorhabditis*^14^, usually in gene families with multiple copies per genome. Innexin homologs have also been identified in the cnidarian *Hydra* and innexin-1 has been localized to gap junctions when expressed with a GFP tag in epithelial cells of *Hydra*^6^.

**Figure 2:**
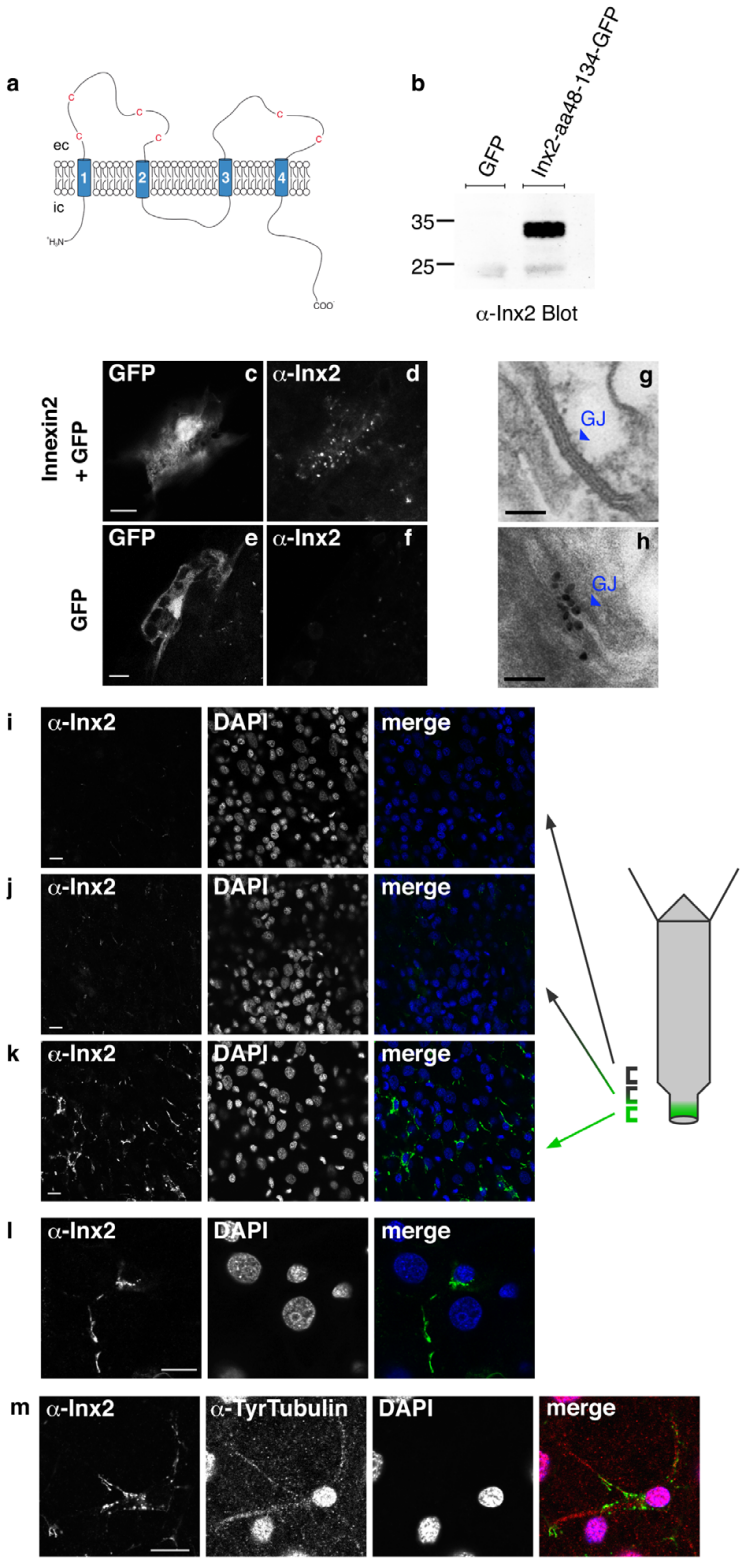
Immunofluorescent staining of innexin-2 in gap junctions in *Hydra*. (a) Schematic drawing of the predicted structure of hydra innexin showing four transmembrane domains and conserved cysteine residues in the extracellular (ec) loops. N- and C-terminus are intracellular (ic). The first extracellular loop of innexin-2 (aa48–134) was used for antibody production. (b) Purified recombinant GFP-tagged innexin-2 (aa48–134) from *E.coli* was detected by the innexin-2 antibody at the appropriate size (ca. 35 kD) in immunoblot. The antibody did not detect GFP. (c–f) Ectopic expression of GFP (used as a transfection marker) and untagged innexin-2 in hydra epithelial cells transfected with the particle gun. To visualize innexin-2 expression, animals were fixed and stained with innexin-2 antibody 48 hours post-transfection. Transfected GFP-expressing epithelial cells displayed a punctate innexin-2 pattern in immunofluorescence when co-transfected with innexin-2 (d). Control animals transfected with GFP only have no detectable innexin-2 signal (f). Scale bar: 10 μm. (g) and (h) Immunogold staining of innexin-2 gap junction in peduncle tissue. (g) TEM image of a typical gap junction (enlarged from Fig. 1A); (h) immunogold staining of innexin-2 gap junction in peduncle tissue. Scale bar: 100 nm. (i–k) Immunostaining of whole animals with innexin-2 antibody revealed innexin-2 positive green spots primarily in the peduncle region (k); more apical areas in the peduncle showed decreased amounts of antibody staining (i and j); the gastric region contained no innexin-2 positive green spots. (l) High magnification image of an innexin-2 positive cell in the peduncle region. The innexin-2 positive green spots were often clustered as strings along nerve processes. Nuclei stained with DAPI. Projections of confocal images covering a depth of 2–3 μm. Scale bar: 10 μm. (m) Co-immunostaining of hydra with innexin-2 antibody and tyrosine-tubulin antibody (Sigma) showed that innexin-2 staining in nerve cells was localized along nerve cell processes. The anti-tyrosine-tubulin staining in nerve cell nuclei is regularly observed but appears to be an artifact (see also Figure 3). Nuclei stained with DAPI. Projections of confocal sections covering a depth of 2 μm. Scale bar: 10 μm.

The *Hydra* genome encodes a family of 17 innexin genes^7^. *Hydra* innexins have about 400 amino acids and are roughly 25% identical to innexins in protostomes. Innexins in Hydra are predicted to have four transmembrane domains forming a protein with two extracellular loops and N- and C-terminal ends located intracellularly (Figure 2A). Four conserved cysteine residues are present in the first loop and 2 cysteines in the second loop.

**Figure 3:**
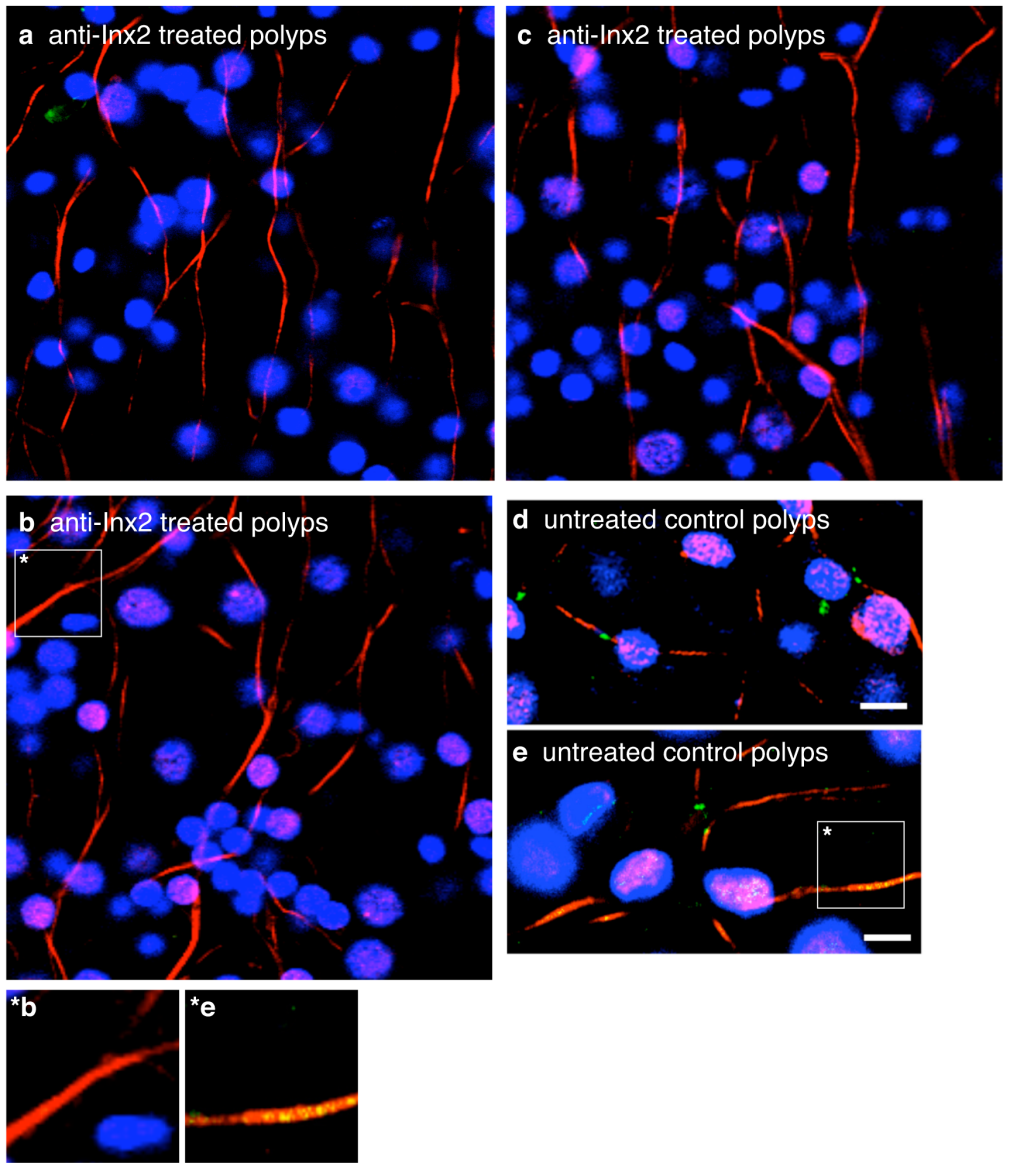
Treatment of *Hydra* polyps with innexin-2 antibody eliminated innexin-2 stained gap junctions in peduncle tissue. (a), (b) and (c) Confocal images of three anti-innexin-2 treated polyps fixed after 3 days and co-immunostained with innexin-2 antibody (green) and tyrosine-tubulin antibody (red). Nuclei are stained with DAPI (blue). (d) and (e) Confocal images of two DMSO treated control polyps. Note that innexin-2 spots (green) are yellow where directly overlapping with strong anti-tubulin (red) stained processes. (*b and *e) Enlargements of single nerve cell processes from b (anti-innexin-2 treated polyps) and e (untreated control polyps). Projections of confocal images covering a depth of 2 μm. Scale bar, 10 μm.

A screen of innexin gene expression in *Hydra* by in situ hybridization showed that innexin-2 is expressed in a population of nerve cells in the lower peduncle of adult polyps (Figure 1C–E). Innexin-2 positive nerve cells were also present in the peduncle of late stage buds (Figure 1C, left side) but not in earlier stage buds (Figure 1C, right side). To localize innexin-2 protein to gap junctions in these cells, we prepared an antibody to the first extracellular domain of innexin-2. The antibody stained recombinant innexin-2 in western blots (Figure 2B) and also in *Hydra* tissue transfected with an innexin-2 gene (Figure 2C–F). To localize innexin-2 in tissue we carried out immunofluorescence staining on whole mounts of fixed *Hydra*. Figure 2 shows confocal images from different positions along the body column. Numerous small spots of innexin-2 staining were observed in the peduncle region but not further up the body column (Figure 2I–K). The spots varied considerably in size and were frequently arranged in chains (Figure 2L) localized along nerve cell processes (Figure 2M and 3E). Individual innexin-2 spots were 300–500 nm in diameter and thus similar in size to the nerve-nerve gap junctions visualized in peduncle tissue by EM (Figure 1A). Quantitative scoring of innexin-2 gap junctions per peduncle led to values of 2000–3000 per peduncle. Since there are about 100 innexin-2 positive nerve cells in the peduncle (Figure 1D–E), we conclude that there are 20–30 gap junctions per nerve cell, i.e. the cells are well connected electrically and could fire synchronously.

To confirm that the innexin-2 spots were localized to nerve cells, we co-stained the animals with an anti-tyrosine-tubulin antibody, which has been shown previously to stain nerve cells in hydrozoans^16^. The images in Figure 2M and 3D–E show that innexin-2 spots are closely associated with the tubulin-stained processes of nerve cells. We also carried out immunogold staining of EM thin sections. Figure 2H shows a patch of gold particles about 100 nm long representing an innexin-2 gap junction (compare to Figure 2G). Such patches were found in peduncle sections, but not in sections from the gastric region.

### Nerve cells expressing innexin-2 gap junctions coordinate contraction of the body column

**Figure 4:**
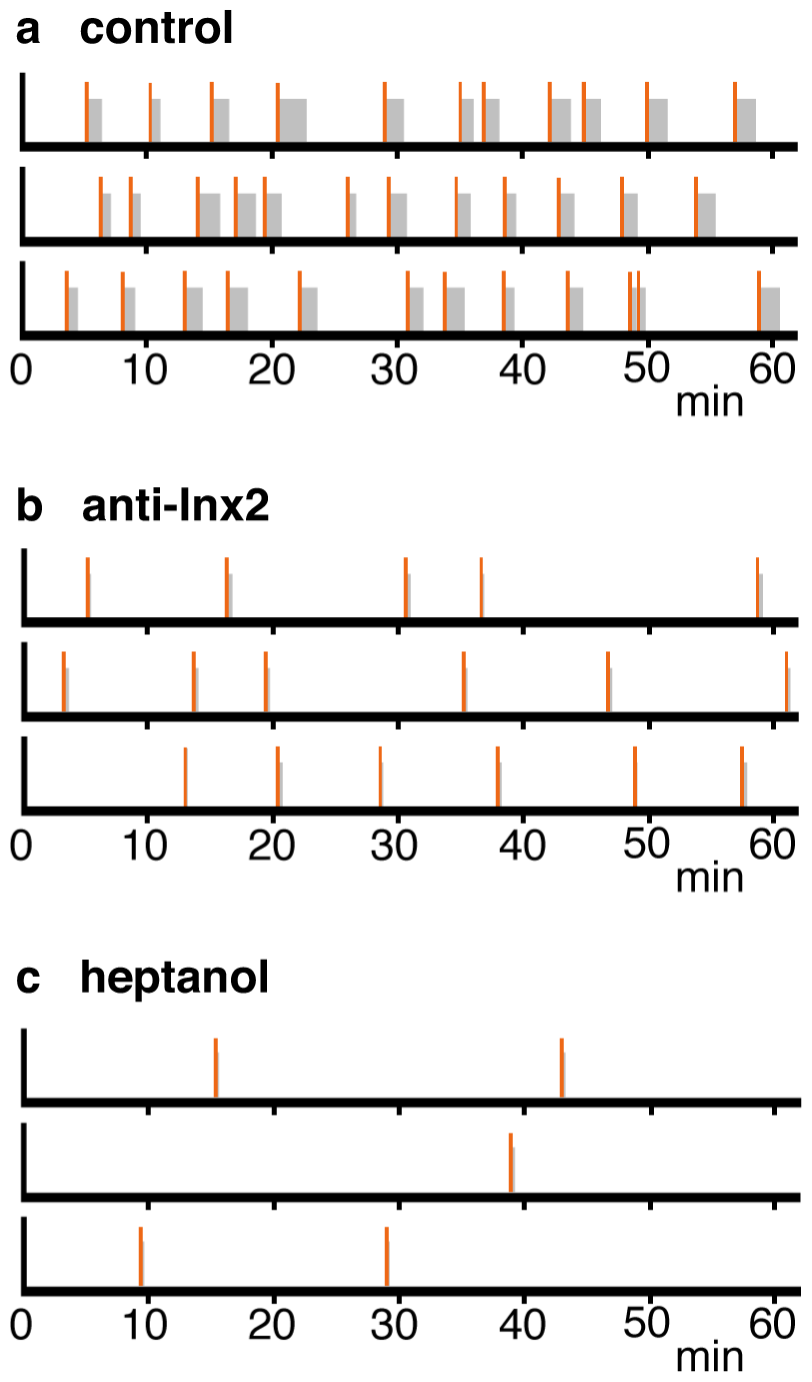
Role of innexin-2 gap junctions in contractile behavior. (a) and (b) Schematic representation of contractile behavior over one hour interval based on time-lapse videos (see Supplemental movie 1 and 2). Data are shown for three individual polyps for each treatment. A single contraction is represented by a vertical bar (red); contraction bursts are represented by a vertical bar (red) followed by a grey bar showing the duration of the burst. Animals were treated with DMSO alone (control) or with DMSO and anti-innexin-2 antibody. (c) Contractile behavior of 3 individual polyps treated with 0.06% heptanol.

*Hydra* polyps in an undisturbed dish in the dark exhibit spontaneous behavior^17^, contracting regularly 7–10 times per hour (see Figure 4A). Each contraction consists of a rapid series of strong contractions of the ectodermal epitheliomuscle cells oriented along the long axis of the polyp. This behavior has been termed a contraction burst and is accompanied by large electrical signals^17^,^18^. Removal of nerve cells from *Hydra* tissue^4^ completely eliminated spontaneous contraction bursts in the body column. Such nerve-free polyps were nearly motionless for hours. Nevertheless, such polyps still responded to strong mechanical stimulation (pinching with forceps) with propagation of electrical signals and coordinated contraction of the body column^4^. Thus contraction of the body column is regulated at two levels: (1) epitheliomuscle cells are connected by gap junctions and can propagate an electrical signal mediating contraction in response to exogenous stimuli and (2) spontaneous initiation of epitheliomuscle cell contraction is controlled by the nervous system, since it is absent in nerve-free animals.

To investigate the role of innexin-2-coupled nerve cells in initiating spontaneous contractions, we treated live animals with innexin-2 antibody in the presence of 0.05% DMSO to facilitate antibody access to tissue^19^. Quite strikingly, after 3 days of treatment innexin-2 gap junctions could no longer be stained in the peduncle region of treated animals (Figure 3A–C). By comparison, DMSO treated control animals contained innexin-2 gap junctions (Figure 3D–E). While we had expected that the innexin-2 antibody would bind to and inhibit gap junctions, it appears to eliminate the junctions altogether. This loss of gap junctions was also observed in EM sections of animals treated with innexin-2 antibody.

To analyze the behavior of antibody-treated animals, we made time-lapse videos of undisturbed animals in red light, which *Hydra* cannot detect^17^ (See Supplemental movie 1 and 2). Figure 4A shows that DMSO-treated control *Hydra* contracted roughly every 5 minutes during a one-hour interval. By comparison, polyps treated with innexin-2 antibody contracted less frequently, about once every 10–12 minutes (Figure 4B). Furthermore, the contraction bursts were markedly shorter than in control animals. These results suggest that nerve cells containing innexin-2 gap junctions are required to initiate and coordinate contraction bursts.

**Figure 5:**
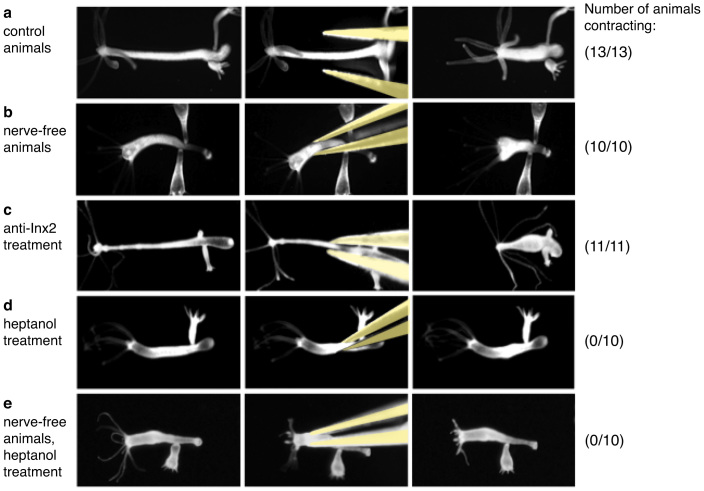
Contractile behavior induced by mechanical stimulation. (a–e) Contraction of the body column was induced by pinching with forceps. Control hydra (a), nerve-free hydra (b), innexin-2 antibody treated hydra (c), heptanol treated normal hydra (d) and heptanol treated nerve-free hydra (e). The number of animals responding with body column contraction is shown on the right.

To confirm that the innexin-2 antibody did not affect the intrinsic ability of the body column to contract, we used a mechanical stimulation assay^4^. When normal *Hydra* were pinched with a pair of forceps, the animals responded with rapid contraction of the body column (Figure 5A). Contraction was also detected when nerve-free animals were pinched (Figure 5B), indicating that nerve cells are not required to coordinate body column contraction. Animals treated with innexin-2 antibody also showed normal contractions after pinching (Figure 5C) indicating that treatment with innexin-2 antibody did not affect the ability of the body column to contract when mechanically stimulated. From these results we conclude that the inhibitory effect of antibody treatment on spontaneous contractile activity is due primarily to inhibition of the neuronal signal initiating contraction.

The above experiments show that nerve-free animals contract when mechanically stimulated by pinching. To confirm the role of gap junctions between epithelial cells in this contractile behavior we conducted further experiments using heptanol to inhibit gap junction communication^20^. In the presence of 0.06% heptanol, contraction of normal animals stimulated by pinching was completely inhibited (Figure 5D). Contraction of nerve-free animals stimulated by pinching was also completely inhibited (Figure 5E). From these results we conclude that signaling between epithelial cells via gap junctions is required for contraction of the body column. In agreement with this conclusion, heptanol-treated animals also showed fewer spontaneous contractions and shorter contraction bursts than untreated control animals (Figure 4C).

## Discussion

The present experiments have identified a population of nerve cells in the peduncle of *Hydra* that are linked by innexin-2 gap junctions. Furthermore, treatment of *Hydra* with an anti-innexin-2 antibody reduced spontaneous contractions of the body column but did not affect the intrinsic ability to contract when mechanically stimulated. Together these results suggest that innexin-2 nerve cells are involved in coordinating the contraction of the body column and thus responsible for the temporal pattern of spontaneous contractions. If this conclusion is correct, then removal of peduncle tissue from *Hydra* should remove the pacemaker and inhibit periodic contraction of the body column. Experiments of Shimizu and Fujisawa^21^ support this conclusion. They found that the frequency of contraction bursts was significantly reduced when peduncle tissue was excised.

More than 50 years ago, electrophysiological experiments carried out by Passano and McCullough^17^,^18^ demonstrated rhythmic potentials (RPs) originating in the peduncle of *Hydra* and showed that they controlled periodic contractions of the body column, so- called contraction bursts. They referred to this activity as a pacemaker but were unable to identify with certainty the cellular basis for the activity. Based on results reported here, innexin-2-positive nerve cells in the peduncle of *Hydra* are the source of the pacemaker activity. These cells are coupled via gap junctions and thus can fire in synchrony^5^. Hence they can signal ectodermal epitheliomuscle cells (the effector cells) around the periphery of the body column and thereby induce synchronous contraction of the body column.

Spontaneous rhythmic behavior is a characteristic feature of the earliest evolving animal phyla^2^,^3^,^17^. Cnidarian medusae in particular show rhythmic swimming movements while polyps exhibit periodic contractile movements. In spite of this superficial similarity, the organization of the nervous system and the control of behavior are fundamentally different between different classes within the phylum Cnidaria^3^. Hydrozoans have nerve cells with well-developed gap junctions^11^ and there is clear evidence of electrically coupled cohorts of nerve cells, which control behavior^22^. By comparison, anthozoans and scyphozoans lack gap junctions and there is no evidence for electrical coupling between nerve cells^3^. This difference in the occurrence of gap junctions is also reflected in the occurrence of innexin genes, which encode the proteins that make up gap junctions. The innexin gene family is highly expanded in both *Hydra* and *Clytia*^7^, the two hydrozoans for which we have extensive EST and genomic databases. By comparison, the sequenced genomes of anthozoans lack clear evidence of innexin genes. Although the genome of the sea anemone *Nematostella*^23^ does contain a single innexin-like sequence^7^, the encoded protein is more closely related to mammalian pannexins, which form hemi-channels and not gap junctions^24^, than to innexins. No innexin related sequences could be identified in the genome of the coral *Acropora digitifera*^25^. Thus, the striking expansion of the innexin gene family in hydrozoans and the extensive formation of gap junctions between different cell types, including nerve cells, appear to be unique to this group of cnidarians and may have enabled development of behavioral activities, which were advantageous for a wide variety of environmental niches^26^.

## Methods

### Strains and culture

A standard wild-type strain of *Hydra magnipapillata* (strain 105)^27^ was used in the present study. Hydra were cultured at 18 ± 1°C as described by Sugiyama and Fujisawa^27^. They were fed on newly hatched brine shrimp nauplii six times a week. Experimental animals were starved for 24 hours prior to use and not fed during the experiment.

### Whole-mount in situ hybridization

In situ hybridization was carried out as described by Grens et al^28^. The concentration of the riboprobe used for hybridization varied from 50 to 200 ng/ml.

### Transmission electron microscopy

Animals were fixed for 12 hours in 2.5% glutaraldehyde and 2% paraformaldehyde in 0.1 M cacodylate buffer (pH 7.4), followed by three washes of 10 minutes each in 0.1 M cacodylate buffer (pH 7.4) containing 4% sucrose. The animals were then postfixed for 60 minutes in ice-cold 1% OsO_4_ in the same buffer and washed three times for 10 minutes each in ice-cold distilled water. Dehydration through a graded series of ethanol solutions was followed by embedding in an Epon-Araldite mixture. Ultra-thin sections (approximately 70 nm) were made, and stained with 2% uranyl acetate followed by 0.4% lead citrate for 5 minutes each. Samples were observed using a JEOL transmission electron microscope (JEM-1010, 80 kV). For immuno-electron microscopic observations, the post-embedding method of Hwang et al^29^ was used.

### Antibody generation

The putative first extracellular loop (amino acids 48–134) of innexin-2 was cloned into the unique NotI and SalI sites of an *Escherichia coli* overexpression vector, pET28a (Novagen, Madison, WI). After verifying the insertion by sequencing, the plasmid was transformed into *Escherichia coli* BL21 (DE3). Overexpression of recombinant innexin-2 was induced with IPTG (isopropyl-1-thio-,3-D-galactoside) and the soluble protein was purified on Ni-NTA agarose (Invitrogen, Carlsbad, CA). Eluted fractions containing recombinant innexin-2 peptide were then subjected to 12% sodium dodecyl sulfate-polyacrylamide gel electrophoresis (SDS-PAGE). A single band of 15 kD was isolated from the gel and delivered to OPERON Biotechnologies, K.K. (Tokyo, Japan) to raise a polyclonal antibody in rabbit. The polyclonal antibody was affinity-purified by protein A-Sepharose.

### Immunofluorescence staining

*Hydra* polyps were relaxed with 2% urethane, fixed with 2% paraformaldehyde in hydra medium for 1 hour and then permeabilised in 0.5% Triton X-100/PBS for 15 minutes. Blocking was performed in 0.1% Triton X-100 and 1% BSA in PBS for 16 hours. Rabbit anti-innexin-2 antibody (see above) and mouse monoclonal anti-Tyrosine-Tubulin antibody (T9028, Sigma) were diluted 1:200 in blocking solution. Alexa488 anti-rabbit and Cy3-coupled anti-mouse antibodies have been used as secondary antibodies. Antibody incubations were performed at room temperature for 1 hour.

Confocal images were acquired with a Leica (Leica Microsystems, Wetzlar, Germany) TCS SP confocal laser-scanning microscope as described previously^30^.

### Behavior experiments

The dynamic movements controlled by the peduncle in hydra were examined under a light microscope (Nikon OPTIPHOT). The observation was recorded with a Hi-band 8-mm formatted video recorder (SONY, EVO-9500A) through a CCD TV camera, which accepted visible and infrared light (Hitachi, KV-26). Experimental animals were placed in a plastic dish containing 10 ml of culture solution and the entire procedure was carried out at 18 ± 1°C. Tentacles were cut off 3 hours before experiments to reduce the movement of animals around the dish. The animals were incubated for 3 days with a 300-fold dilution of the antibody in hydra culture medium containing 0.05% DMSO. Control animals were also treated with 0.05% DMSO for 3 days. These solutions were changed every 12 hours. In some experiments, animals were treated with heptanol (0.06%; Wako) and, after incubation for 1 hour, analyzed for contractile activity.

### Plasmids

Full-length innexin-2 was amplified from *Hydra* cDNA by PCR and sub-cloned in the Hydra EGFP expression vector by using the Nhe1 and Sma1 restriction sites^31^. Site-directed mutagenesis was used to introduce two adjacent stop-codons in between the innexin-2 open reading frame and the GFP sequence. This resulted in expression of untagged full-length innexin-2 after transfection into *Hydra* cells.

*Hydra* innexin-2 amino acids 48–134 were cloned into pRSet5D, containing the GFP coding sequence^32^ and subsequently expressed in *E. coli* BL21 (DE3).

### Transfection of Hydra cells

*Hydra* cells were transfected with a particle gun as described elsewhere^31^.

## Author Contributions

Y.T., A.W., A.B., C.N.D. and T.G. planned the experiments. Y.T., J.S.H., A.W. and H.S. performed the experiments. Y.T., A.W., C.N.D. and T.G. wrote the paper.
